# Intraoral Sebaceous Carcinoma: Case Report of a Rare Tumor Emphasizing the Histopathological Differential Diagnosis

**DOI:** 10.1155/2018/3054931

**Published:** 2018-07-09

**Authors:** Manveen Kaur Jawanda, R. V. Subramanyam, Harshaminder Grewal, Chitra Anandani, Ravi Narula

**Affiliations:** ^1^Department of Oral Pathology, Luxmi Bai Institute of Dental Sciences, Patiala, Punjab, India; ^2^Department of OMFS and Diagnostic Sciences, College of Dentistry, King Faisal University, Al-Ahasa 31982, Saudi Arabia; ^3^Department of Oral Pathology, Desh Bhagat Dental College, Mandi Gobindgarh, Punjab, India; ^4^Department of Oral and Maxillofacial Surgery, Guru Nanak Dev Dental College, Sunam, Punjab, India

## Abstract

**Background:**

Sebaceous carcinoma (SC) is an uncommon cutaneous malignancy, usually occurring predominantly in the eyelids and only occasionally involving the oral cavity. Sebaceous carcinoma (SC) is a rare malignancy. Only 10 cases of sebaceous carcinoma of the oral cavity have been reported so far.

**Case Presentation:**

A 40-year-old female presented with a mass on the left side of the middle third of the face. Radiographic findings were inconclusive. Resection of the mass was consistent with the diagnosis of primary sebaceous carcinoma.

**Conclusion:**

Intraoral sebaceous carcinoma is uncommon. Due to its varied clinical appearance and presence of a diverse histopathologic appearance, the diagnosis is quite often confounding and elusive. Hence, it is imperative to familiarize oneself about various aspects of this rare tumor for earlier diagnosis, to improve the chances of patient's survival.

## 1. Introduction

Sebaceous carcinoma has been defined by the WHO as “a malignant tumor composed of sebaceous cells of varying maturity that are arranged in sheets and/or nests with different degrees of pleomorphism, nuclear atypia, and invasiveness” [1].

Sebaceous carcinoma is an aggressive, uncommon, cutaneous tumor first described by Allaire in 1891. This tumor is thought to arise from sebaceous glands in the skin and thus may arise anywhere on the body where these glands exist [2]. Sebaceous carcinoma predominantly occurs in the skin of the eyelid, face, neck, and scalp [3]. Extraocular noncutaneous sebaceous carcinoma mainly involves the major salivary glands. Primary sebaceous carcinoma of oral cavity is rare [4]. It is thought to arise from Fordyce granules or salivary gland elements [5]. Oral sebaceous carcinoma can be a diagnostic challenge for the clinicians as well as the pathologist. Because oral sebaceous carcinoma presents most commonly as an asymptomatic nonencapsulated nodule, diagnosis and treatment therapy tend to be delayed because it is frequently mistaken for more common benign entities. In addition to its varied clinical appearance, the presence of a diverse histologic appearance may delay the diagnosis or result in a misdiagnosis. To our knowledge, only ten cases of intraoral sebaceous carcinoma have been reported in the literature [6]. The current report describes another case of oral sebaceous carcinoma, indicating the need for comprehensive histopathological differential diagnosis.

## 2. Case Presentation

**Figure 1:**
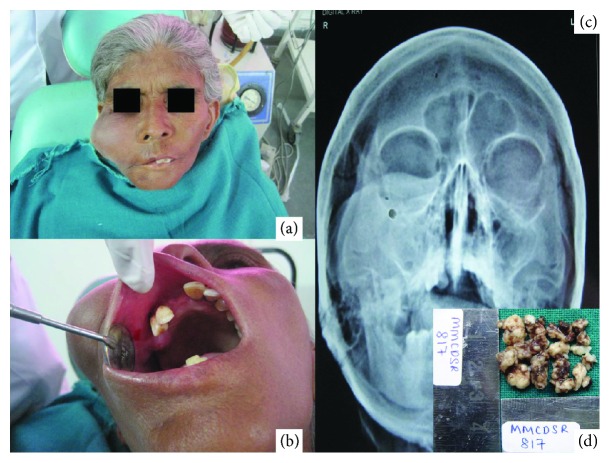
(a) Clinical and (b) intraoral picture showing an intact buccal mucosa. (c) Water's view radiograph of the patient. (d) Gross incisional tissue.

A 40-year female reported with a swelling on the right side, involving the middle third of the face, since 1 year. The swelling was firm in consistency, nontender, and of approximately 5 × 4 cm, extending superoinferiorly from the infraorbital ridge to 2 cm above the inferior border of the mandible and anteroposteriorly from the right corner of the mouth to 1.5 cm anterior to the tragus (Figure 1(a)). The borders of the swelling were diffuse, and the skin overlying the swelling was normal in color. The swelling was mobile with no ulceration of the overlying skin. Intraoral examination revealed no obvious swelling with intact oral mucosa, and scattered foci of Fordyce's spots were seen on the buccal mucosa (Figure 1(b)). Water's view of the skull showed impression of the soft tissue swelling in the right cheek area (Figure 1(c)). The hemoglobin level was 7.5 gm/dl (anemic), and other routine hematological findings were within normal limits. Chest radiograph showed no abnormality. No palpable lymph nodes were found. A definite clinical diagnosis was not possible, and an incisional biopsy (Figure 1(d)) was taken from the buccal mucosa and subjected to histopathological examination.

**Figure 2:**
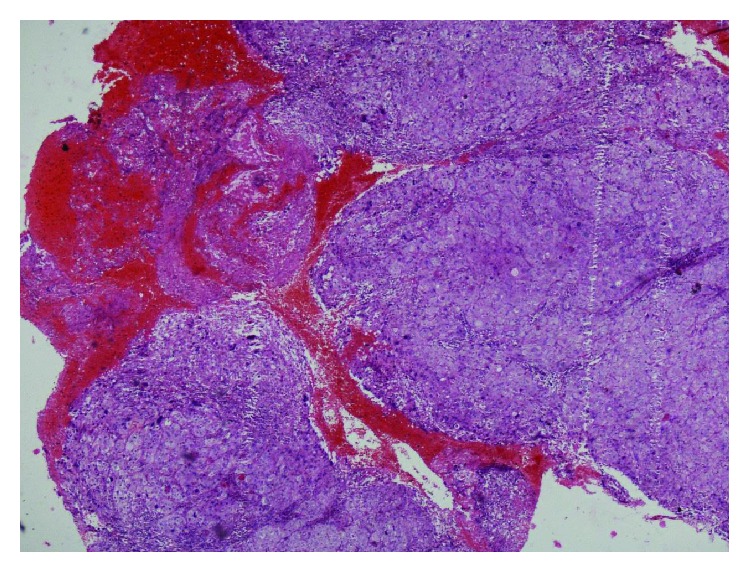
Pattern of asymmetry, a lack of circumscription with pushing or locally infiltrating margins (H and E stain; original magnification, 4x).

**Figure 3:**
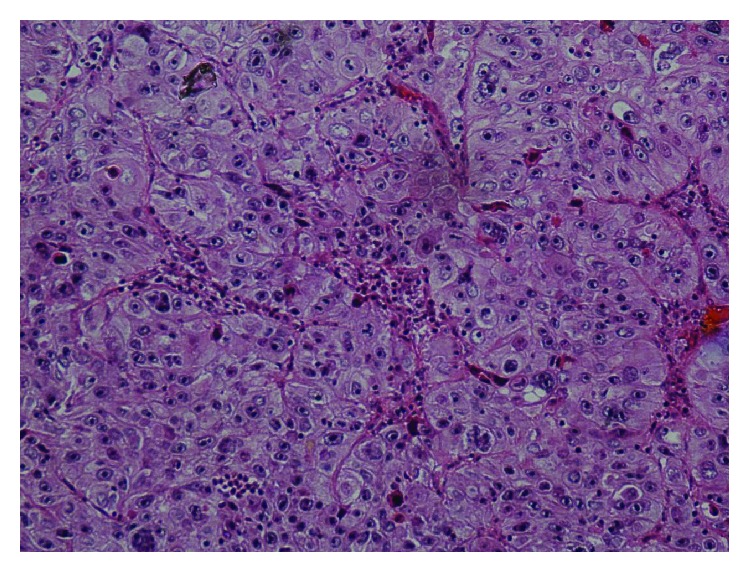
Large nests or lobules of neoplastic cells with squamous appearance, separated by scant stroma (H and E stain; original magnification, 10x).

**Figure 4:**
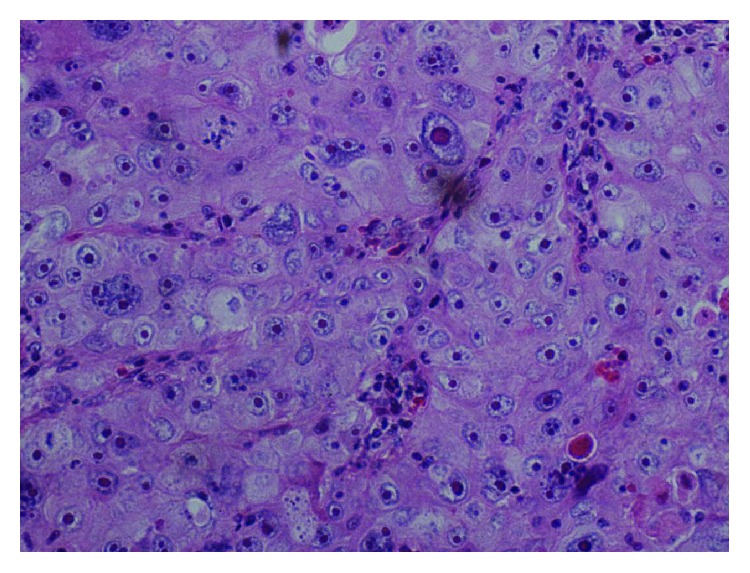
Neoplastic cells had large vesicular nuclei with prominent nucleoli. Cellular and nuclear pleomorphism with few nuclei showing multilobation. Scattered typical and atypical mitotic figures seen (H and E stain; original magnification, 20x).

**Figure 5:**
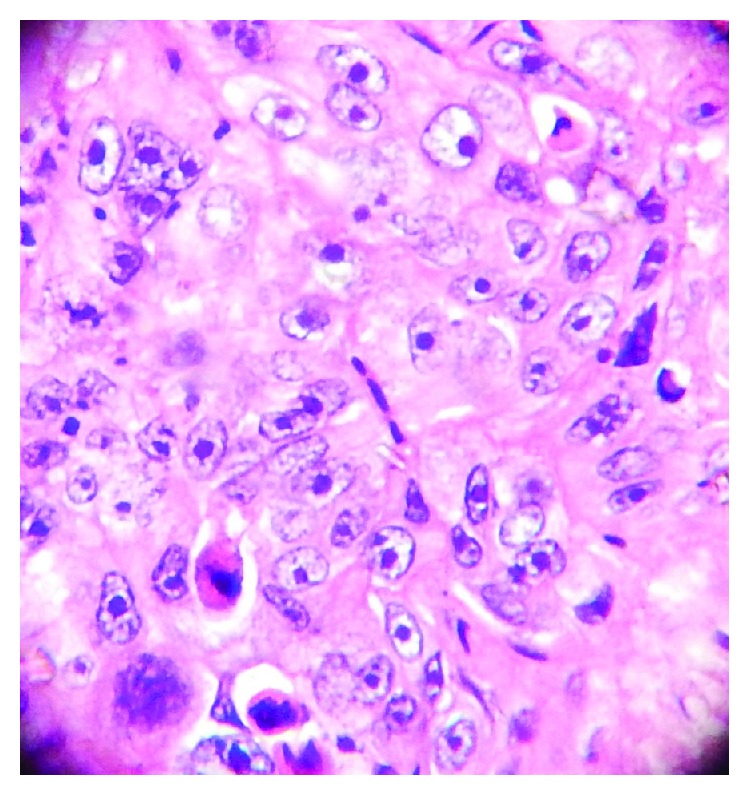
Sheets of multivacuolated/vesiculated cells having squamoid appearance, multilobation of some nuclei, high mitotic rate with abnormal mitosis (H and E stain; original magnification, 100x).

**Figure 6:**
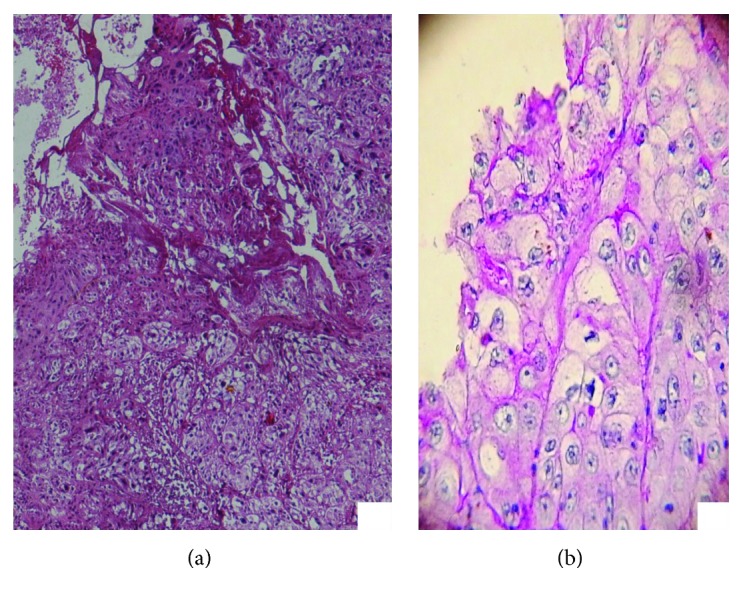
Photomicrograph showing lobules composed of clear tumor cells with foamy cytoplasm exhibiting absence of any mucin on PAS stain. (a) H and E stain; original magnification, 10x. (b) PAS stain; original magnification, 40x.

**Figure 7:**
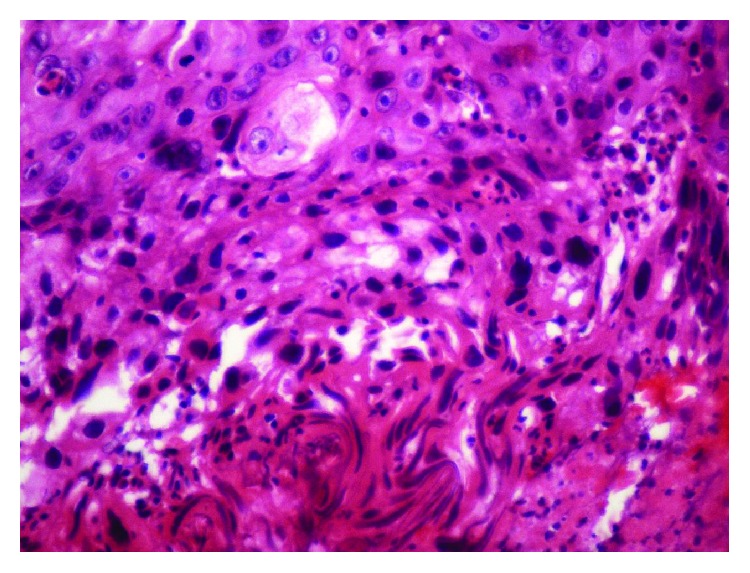
Variable number of smaller, darkly staining basaloid cells with oval-shaped nuclei and scant cytoplasm (H and E stain; original magnification, 40X).

Microscopically, the tumor mass appeared to be located in the deeper mucosa with pushing margins of tumor nests (Figure 2). The tumor was composed of large nests of neoplastic cells with squamous appearance, separated by scanty stroma (Figure 3). The neoplastic cells had large vesicular nuclei with prominent nucleoli. Cellular and nuclear pleomorphism with few nuclei showing multilobation was seen, along with typical and atypical mitotic figures (Figures 4 and 5). The sebaceous nests were composed of clear tumor cells with foamy cytoplasm exhibiting absence of mucin on periodic acid-Schiff (PAS) stain (Figure 6). In contrast, a variable number of smaller, darkly staining basaloid cells with oval-shaped nuclei and scant cytoplasm were also seen (Figure 7). A final diagnosis of sebaceous carcinoma was accorded based on the histopathological features. The patient was further advised for a full body scan and referred to an oncologist for further treatment.

## 3. Discussion

The diagnosis of oral sebaceous carcinoma, “a benign-appearing” malignant neoplasm, remains challenging both clinically and histopathologically. Any anatomic site that contains sebaceous glands may potentially give rise to neoplasms exhibiting sebaceous differentiation. Fordyce's spots represent ectopic sebaceous glands in the oral cavity and are commonly found in the buccal mucosa, upper lip, retromolar trigone, anterior tonsillar pillar, soft palate, and gingiva [7]. Although approximately 80% of the adult populations have clinically evident sebaceous glands in the oral mucosa, only ten cases of sebaceous carcinoma have been documented until now. Intraoral sebaceous carcinoma was first reported by Damm et al. in 1991 (Table 1, case 1) [4]. Since then, another 9 cases were reported in the literature (Table 1) [4–6, 8–14].

The origin of sebaceous carcinoma in the oral cavity is still unclear. It may arise from intraoral minor salivary glands, parotid duct, or Fordyce granules [4, 5]. In the current case, there was confusion about the possible origin of the neoplasm. Several possible origins were thought on the basis of close association of various structures with the tumor. As the tumor presented as a swelling on the middle third of the face with normal overlying skin and an intact buccal mucosa intraorally, with scattered foci of Fordyce's spots, there was a confusion regarding the primary site. On the basis of close association of the swelling to the eye and ear, the lower eyelid, external auditory canal, preauricular lymph nodes, and parotid gland were thought to be the primary sites. The patient was therefore submitted to instrumental examination (eye, ear) to evaluate the possible origin, all with a negative result. No preauricular lymph node enlargement was found; furthermore, no evidence of appendageal structures within the biopsy specimen was seen, thus ruling out the possibility that the tumor originated from the skin of the cheek or lymph node. Therefore, based on these observations, it was concluded that the primary site of the tumor was the buccal mucosa. The presence of Fordyce granules in the area of involvement suggests that the tumor may have arisen from the malignant transformation of these ectopic sebaceous glands [11]. Alternatively, sebaceous differentiation of Stensen's duct could also be considered a probable etiology due to the inclination towards the buccal region [14].

Histologically, sebaceous carcinoma shows quite a range of differentiation, ranging from obviously multivacuolated epithelium to basaloid or squamoid populations of cells with more occult cytoplasmic lipid content [15]. Hence, it is imperative to differentiate an intraoral sebaceous carcinoma from basal cell carcinoma with sebaceous differentiation, clear cell as well as basaloid squamous cell carcinoma with hydrophilic swelling, metastatic clear cell renal carcinoma, and salivary gland malignancies such as mucoepidermoid carcinoma, solid-type adenoid cystic carcinoma, basal cell adenocarcinoma, and salivary duct carcinoma [4, 13].

**Figure 8:**
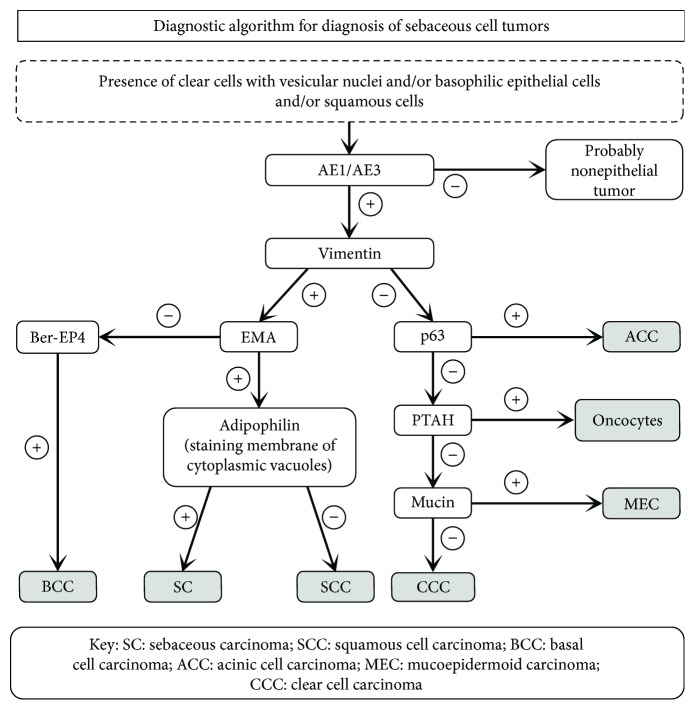
Diagnostic algorithm depicting the use of immunohistochemistry (IHC) to logically arrive to the diagnosis of sebaceous carcinoma (SC).

According to Plaza et al. [16], histology remains the gold standard for the diagnosis of SC, and they suggested that the immunohistochemical assessment for epithelial markers and lipid droplet-associated proteins is a helpful diagnostic adjunct and that immunostaining for epithelial markers should be performed once careful standard microscopic evaluation has taken place. If these results are nonconclusive for SC, the prospective diagnosis can be confirmed by a lipid droplet-associated protein, such as adipophilin (Figure 8) [16].

We did not perform IHC in our case because the diagnosis was quite obvious. Periodic acid-Schiff (PAS) stain was negative, confirming that the vacuolated clear cells were neither mucus cells nor glycogen-rich squamous cells, thus ruling out the possibility of a carcinoma arising from oral epithelium or salivary epithelium. Moreover, clear cell squamous cell carcinoma is characterized by areas of squamous differentiation with foci of keratinization and keratin pearls, which were absent in our case. In addition, basaloid squamous cell carcinoma was also excluded due to the absence of comedonecrosis, hyalinization of the stroma, or microcyst formation.

Furthermore, we observed a variable number of smaller, darkly staining, basaloid cells with oval-shaped nuclei and prominent nucleoli. These cells are thought to represent undifferentiated sebaceous cells [17], thus giving a basaloid cytological appearance; however, peripheral palisading pattern of the basaloid cells and retraction artifacts between the mucinous stroma and the tumor nests, characteristic of basal cell carcinoma, were absent. Also, clear cells with foamy-bubbly cytoplasm or starry nuclei, typical of sebaceous cells, were seen in our case. A diagnosis of intraoral basal cell carcinoma (with sebaceous differentiation) was, therefore, excluded.

In the current case, due to the presence of large nests of polygonal tumor cells with an optically clear cytoplasm, metastatic clear cell renal carcinoma was also thought as one of the differential diagnoses. This tumor is characterized by tumor cells arranged in nests and separated from each other by extensive rich network of delicate sinusoidal vascular channels. The tumor cells are generally large and polygonal, having a distinct cell membrane as if drawn by a “pencil” and an optically clear cytoplasm. This clear appearance of the cytoplasm of clear cell renal carcinoma is due to the presence of abundant glycogen and neutral lipids but not mucin [18]. However, PAS negativity in our case excludes the possibility of this neoplasm.

Besides, benign tumors, including sebaceous adenoma, could not be considered in the histological differential diagnosis due to the infiltrative pattern and cytological features associated with sebaceous carcinoma. The presence of foci of cells with cytoplasmic microvacuoles and atypical scalloped nuclei confirmed the diagnosis of sebaceous carcinoma and ruled out the abovesaid malignant neoplasms.

The treatment of choice for sebaceous carcinoma is surgery, with complete excision verified by negative margins. Radiotherapy is used if metastatic disease and/or a high risk of recurrence are present. Multiagent chemotherapy has been used to treat recurrent disease [19]. Nevertheless, an increased proclivity for local recurrence and metastasis calls for a long-term follow-up of the affected patients [11].

## 4. Conclusion

Sebaceous carcinoma is a very aggressive, rare tumor which is generally not considered in the differential diagnosis of tumors arising from a site such as the buccal mucosa. This often leads to a delay in treatment. We emphasize the need to generate awareness about this rare entity occurring at unusual sites to expedite the patient's survival.

## Figures and Tables

**Table 1:** Clinical findings of reported cases of intraoral sebaceous carcinoma.

	Reference	Age/sex	Anatomic site	Size (cm)
1.	Damm et al. [4]	53/M	Left buccal mucosa	3
2.	Abuzeid et al. [8]	11/F	Left buccal mucosa	3
3.	Liu et al. [5]	68/M	Right buccal mucosa	2.5
4.	Li et al. [9]	78/M	Left buccal mucosa	3.5
5.	Handschel et al. [10]	80/F	Anterior floor of the mouth	1.5
6.	Alawi and Siddiqui [11]	66/M	Left upper labial mucosa	1.5
7.	Gomes et al. [12]	55/M	Right floor of the mouth	Not known
8.	Wang et al. [6]	50/M	Left buccal mucosa	4.6
9.	Oshiro et al. [13]	66/M	Dorsum of tongue	2.5
10.	Rowe et al. [14]	76/M	Gingival mucosa, with metastasis to the lung and subcutis	3
11.	Present case (2017)	40/F	Right buccal mucosa	5
